# Feed cost efficiency in robotic milking systems: an analysis of revenue minus feed cost across dairy cow groups in southern Brazil

**DOI:** 10.1007/s11250-026-05105-7

**Published:** 2026-06-08

**Authors:** Caroline Braga Andelieri, Marcele Sousa Vilanova, Adilson Lemos Rezende, Damiano Cavallini, Giovanni Buonaiuto, Tiago Bordin

**Affiliations:** 1 Postgraduate Program in Dairy Cattle Nutrition, CESURG Marau Faculty, Marau, Rio Grande do Sul, Brazil; 2 University of Caxias do Sul – UCS, Caxias do Sul, Rio Grande do Sul, Brazil; 3 Department of Veterinary Medical Sciences (DIMEVET), Alma Mater Studiorum - University of Bologna, Ozzano dell’Emilia, Bologna, Italy; 4 Agronomy Course, CESURG Marau Faculty, Marau, Rio Grande do Sul, Brazil

**Keywords:** Feed efficiency, Production cost analysis, Economic performance indicator, Robotic milking systems

## Abstract

This study aimed to evaluate the economic impact of grouping strategies on Revenue Minus Feed Cost (RMFC) in a guided-flow Automatic Milking System (AMS) in southern Brazil. RMFC was used as the main economic indicator and was calculated as the difference between milk income and feed cost per cow. This retrospective observational study was conducted on a commercial dairy farm using routine production and economic records from 76 Holstein cows managed according to the farm routine as a single group in 2022 and as high- and low-production groups in 2023. Daily milk yield, feed costs, and milk prices were recorded from February to August. Data were analyzed considering management group and month, using ANOVA and Tukey’s test at the 5% significance level. RMFC was significantly affected by group type, feed costs, and milk price. The high-production group (LA) presented the highest feed efficiency (FE) and RMFC, despite requiring higher nutritional investment. The low-production group (LB) had reduced economic performance, especially in the final months of lactation. Under the evaluated farm conditions, the grouped strategy was associated with more targeted feeding management and with differences in technical and economic performance among groups. This study contributes to the economic evaluation of grouping strategies in AMS under commercial conditions, using RMFC and FE as technical-economic indicators.

## Introduction

Milk is one of the main agricultural commodities worldwide, both for its nutritional value and economic relevance. In Brazil, according to the Instituto Brasileiro de Geografia e Estatística (IBGE 2025), the national cattle herd reached 238.6 million head in 2023, maintaining the country as the holder of the largest commercial herd in the world. Formal milk production totaled approximately 25.38 billion liters in 2024, a 3.1% increase compared with the previous year. Dairy activity is present in about 98% of Brazilian municipalities, predominantly supported by small and medium-sized farms, and generates nearly four million direct and indirect jobs.

The growing demand for productivity, combined with a shortage of skilled labor, has driven technological advances in the dairy sector in recent decades (Giannone et al. 2025; Lamanna et al. 2025a, Lamanna et al.2025b, Lamanna et al.2025c; Cavallini et al. 2025). One milestone of this transformation was the introduction of AMS, first implemented commercially in the Netherlands in 1992 (Svennersten-Sjaunja and Pettersson 2008). The global adoption of AMS has accelerated, and the robotic milking market was estimated to reach USD 4.31 billion by 2027 (Lage et al. 2024).

These systems transformed traditional milking practices by allowing cows to be milked automatically without human intervention. Reported benefits include increased milking frequency, continuous monitoring of individual indicators, improved animal welfare, and encouragement of generational succession through the technification of dairy operations (Jacobs and Siegford 2012; Tse et al. 2018). However, efficient use of this technology demands specific management adaptations and raises new questions regarding the effectiveness of adopted strategies.

A key aspect of herd management in AMS is the control of cow traffic, how animals access the milking robot. Two main models are adopted: free-flow and guided-flow. This study was conducted under a guided-flow configuration, which provides greater predictability and organization in herd management (Solano et al. 2022).

In addition to traffic control, AMS technology enables individualized feeding by providing concentrate feed during milking according to each cow’s daily milk yield (Yan et al. 2009; Rongwei 2024). This precision feeding approach eliminates the need for production grouping, since each animal is managed as an autonomous unit. Consequently, many AMS herds operate without physical grouping, unlike conventional systems (Sharipov et al. 2020).

Nevertheless, studies on conventionally managed herds have shown that grouping cows according to productivity or lactation stage can improve FE and productive performance (Cabrera and Kalantari 2016). Hence, an important question arises: Is it still worthwhile to segment cows by production level in technologically advanced environments? Few studies have explored this strategy in AMS herds, particularly from an economic perspective.

This knowledge gap motivated the present study, which aims to evaluate the economic impact of production grouping in a guided-flow AMS (“Milk First” configuration). Two management strategies were compared: a single group and a divided system (high- and low-production). Performance was measured using the RMFC indicator. This study investigates whether production grouping can improve economic performance in a guided-flow AMS under commercial conditions.

In this context, RMFC is a useful economic metric for assessing dairy profitability. It represents the daily balance between revenue from milk sales and feed cost per cow, providing a practical and objective measure of herd economic return. By integrating productive and financial indicators, this index supports more accurate analyses of management and nutritional strategies, especially in automated systems, where operational costs are high and profit margins are sensitive to market fluctuations.

## Material and methods

This retrospective observational study was based on routine farm records from a commercial dairy herd. No experimental treatments were imposed by the researchers. The study describes and compares two herd management strategies adopted by the farm in consecutive years under commercial conditions: a single-group strategy in 2022 and a grouped strategy based on milk yield in 2023. Descriptive reports and field observations were used only for contextual purposes and were not used to generate the quantitative variables analyzed in the study.

This study was conducted using productive and economic data from a commercial dairy farm, located in Nova Bassano, Rio Grande do Sul, Brazil (28°43′26″S, 51°42′18″W, 563 m above sea level). The local climate is classified as humid subtropical (Cfa) according to Beck et al. (2018). The analyzed period covered February to August of 2022 and 2023.

The dataset included information on 76 Holstein cows selected from a total herd of 100 animals, with an average body weight of 700 ± 20 kg, milk yield of 40 ± 2 L/cow/day, mean age of 46 ± 2 months, and 230 ± 5 days in milk. The evaluated groups corresponded to the herd organization adopted by the farm in each year, based on the cows included in the production records for the analyzed period. The animals were housed in two barns with mixed structure (metal and masonry), measuring 50 × 20 m (Barn 1) and 70 × 20 m (Barn 2). Both barns operated under a Compost Barn system with *Pinus elliottii* sawdust bedding. Milking was performed exclusively using DeLaval® VMS™ V300 robots operating under the guided-flow “Milk First” configuration.

There was no direct researcher intervention in animal handling or farm management. All procedures were carried out by the farm management team as part of routine operations. Consequently, this study is classified as a secondary data analysis, for which approval from the Animal Use Ethics Committee (CEUA) was not required.

### Data collection

Quantitative data used in this study were obtained from routine farm records. The main data sources included automated records from the DeLaval DelPro™ system, which provided information such as daily milk yield, milking frequency, and concentrate intake; the farm’s internal control spreadsheets, which consolidated zootechnical and operational data; and purchase invoices for feed inputs, which were used to calculate diet costs.

Descriptive reports provided by the farm’s technical managers were used only to contextualize the herd organization and feeding strategies adopted in 2022 and 2023. These descriptive sources were not used to generate the quantitative variables analyzed in the study. Because they were used solely for contextual purposes, no formal validation procedure was performed.

### Experimental design and group management

In 2022, all 76 cows were managed as a single group (LU), reflecting the routine herd management adopted by the farm at that time. Under this strategy, cows were not separated according to production level, and all animals received the same partial mixed ration. The average milk yield of this group was 40 ± 2 L/cow/day.

In 2023, the farm adopted a different herd management strategy and organized the cows into two groups according to daily milk yield: a high-production group (LA), consisting of 38 cows averaging 48 ± 0.5 L/cow/day, and a low-production group (LB), consisting of 38 cows averaging 34 ± 0.7 L/cow/day. Under this strategy, feed supply was adjusted according to the productive level of each group in order to improve the economic efficiency of feeding management. Thus, the herd organization described in this study reflects the management routine adopted by the farm in each evaluated year rather than an experimental allocation imposed by the researchers.

Both groups were maintained in Compost Barn housing and milked automatically by two DeLaval® VMS™ V300 robots operating under guided-flow management. Each cow had individual identification, which granted access authorization to the robots.

The diet followed a Partially Mixed Ration (PMR) model and was distributed three times daily using an IPACOL® VFTM 6.0 feed mixer wagon. During milking, concentrate feed was supplied in proportion to each cow’s milk yield, at a ratio of 1 kg per 7 L of milk.

Water was provided *ad libitum* through eight automatic troughs per barn. The Compost Barn bedding was turned twice daily (10:00 a.m. and 5:00 p.m.) using SOLUMONT® equipment.

Daily milk production was recorded in kg/cow/day and converted to liters using Eq. (1):

1 $$\rm{Milk\,{\text{ }}volume}{\text{ }}\left( L \right){\text{ }} = {\text{ }}\rm{Milk\,{\text{ }}mass}{\text{ }}\left( {kg} \right)/{\text{ }}\rm{Milk\,{\text{ }}density}{\text{ }}\left( \rm{{kg/L}} \right)$$

where milk density was assumed as 1.03 kg/L, following standard literature values for bovine milk (Watson and Tittsler 1961).

### Diet composition and cost assessment

The LU diet included whole-plant corn silage and wheat straw (total forage), as well as dried distillers grains (DDG), soybean hulls, a homemade feed mix (ground corn, soybean meal, minerals, vitamins, sodium bicarbonate, and a mycotoxin binder), commercial concentrates (robot feed and pro-peak), high-moisture corn grain, whole cottonseed and added water. Diet costs were monitored monthly (Table 1). The nutritional composition of the diet is described in Table 2 (NASEM, 2021).

**Table 1:** Composition and daily feed cost of the single group (LU) diet in August 2022

Feed ingredient	Quantity (kg/cow/day, as-fed basis)	Unit price (R$/kg)	Daily cost (R$/cow)
Total forage			
Whole-plant corn silage	26.00	0.50	13.00
Wheat straw	0.20	0.55	0.11
Total concentrate			
Dried Distillers Grains	1.00	2.55	2.55
Soybean hulls	3.17	1.20	3.80
Homemade feed mix	5.00	2.55	12.75
Robot feed (average)	2.20	2.44	5.37
Pro-peak feed (average)	2.10	3.10	6.51
High-moisture corn grain	3.00	1.20	3.60
Whole cottonseed	1.60	2.00	3.20
Water added	9.00	–	–
Total	57.27	–	R$ 53.78

**Table 2:** Nutritional composition of diets for the single (LU), high (LA) and low (LB) production groups

Nutrient^1^	LU	LA	LB
Dry Matter (DM), %	46.9	44.3	42.0
Forage, % DM	41.5	46.0	56.5
CP, % DM	17.3	21.2	17.8
ME, Mcal/kg	2.71	2.69	2.67
MP, % DM	9.85	11.13	10.28
NEL, Mcal/kg	1.79	1.78	1.76
RDP, % DM	11.7	14.2	12.2
RUP, Base, % DM	5.6	7.0	5.6
Digestible RUP, % DM	4.5	5.8	4.6
ADF, % DM	21.4	18.2	18.6
NDF, % DM	33.5	28.8	29.7
ADF/NDF, Ratio	0.64	0.63	0.63
Forage NDF, % DM	17.3	18.4	21.8
Starch, % DM	25.4	23.2	27.6
WSC, % DM	5.3	6.1	5.7
Ash, % DM	7.8	9.4	8.6
Fatty Acids, % DM	3.74	3.66	2.96
Ca, % DM	0.64	0.80	0.73
P, % DM	0.42	0.49	0.46
Mg, % DM	0.36	0.43	0.39
K, % DM	1.15	1.29	1.20
Na, % DM	0.50	0.73	0.61
Cl, % DM	0.20	0.20	0.22
S, % DM	0.24	0.27	0.23
DCAD, mEq/kg	308	424	369

^1^Forage, % DM: Percentage of forage in the diet on a dry matter basis; CP: Crude protein; ME: Metabolizable energy; MP: Metabolizable protein; NEL: Net energy for lactation; RDP: Rumen degradable protein; RUP: Rumen undegradable protein; ADF: Acid detergent fiber; NDF: Neutral detergent fiber; WSC: Water-soluble carbohydrates; Ca: Calcium; P: Phosphorus; Mg: Magnesium; K: Potassium; Na: Sodium; Cl: Chloride; S: Sulfur; DCAD: Dietary cation–anion difference

The LA and LB diets (Table 3) differed in formulation and ingredient proportions from the LU diet, being adjusted to the nutritional requirements associated with each production level and to feed availability in the respective year.

**Table 3:** Composition and daily feed cost of the high-production (LA) and low-production (LB) group diets in August 2023

Feed ingredient	LA – Quantity (kg/cow/day, as-fed)	Unit price (R$/kg)	Daily cost (R$/cow)	LB – Quantity (kg/cow/day, as-fed)	Unit price (R$/kg)	Daily cost (R$/cow)
Total forage						
Whole-plant corn silage	30.0	0.35	10.50	30.0	0.35	10.50
Wheat silage	6.00	0.21	1.26	6.00	0.21	1.26
Wheat straw	0.70	1.00	0.70	0.30	1.00	0.30
Total concentrate						
Dried Distillers Grains (DDG)	2.00	2.25	4.50	0.507	2.25	1.14
Toasted soybean grain	1.40	2.90	4.06	0.50	3.00	1.50
Soybean hulls	1.00	1.15	1.15	0.50	1.15	0.57
Homemade feed mix	8.00	2.856	22.85	5.00	3.00	15.00
Robot feed (average)	1.90	2.18	4.14	2.50	2.18	5.45
Pro-peak feed (average)	2.20	2.78	6.11	1.50	2.78	4.17
Urea	–	–	–	0.02	3.00	0.06
Water added	9.00	–	–	5.00	–	–
Total	62.20	–	R$ 55.27	51.82	–	R$ 39.95

The LA diet contained higher proportions of energy- and protein-rich ingredients. Toasted soybean grain was included to meet the higher protein and energy demands of high-yielding cows. Conversely, the LB diet, had lower inclusion rates of these ingredients due to the reduced production potential of this group.

Both diets adopted in 2023 excluded whole cottonseed and high-moisture corn grain, introducing wheat silage as an alternative source of effective fiber. The homemade feed mix was supplemented with urea as a source of non-protein nitrogen (NPN; Table 2).

These formulations illustrate the nutritional differentiation implemented according to productive potential and resource availability on the farm. Daily feed costs per cow for both groups are shown in Tables 1 and 3.

Based on the information presented in Tables 1 and 3, the average daily diet cost per cow was calculated for LA and LB in 2023, and for LU in 2022. Monthly variations reflected supplier pricing and formulation adjustments throughout the analyzed period. The consolidated data are presented in Table 4.

**Table 4:** Daily feed cost per cow (R$) for the high-production (LA), low-production (LB), and single (LU) groups

Month	LU cost (R$)	LA cost (R$)	LB cost (R$)
February	55.41	51.88	39.12
March	55.41	51.88	39.12
April	55.41	51.88	39.12
May	56.78	51.88	39.12
June	54.07	63.49	49.93
July	54.07	57.30	39.95
August	53.78	55.27	39.95
Average	54.99	54.79	40.90

These monthly cost values were used to calculate the main productive and economic indicators described in the following section.

### Zootechnical and economic indicators evaluated

The main economic indicator evaluated was RMFC, calculated for each cow based on average daily milk yield (L/cow/day). Milk production data were recorded daily by sensors in the AMS and stored in the DeLaval DelPro™ software, originally expressed in kg/cow/day and subsequently converted to L/cow/day using Eq. (1). Milk price (R$/L) was obtained from monthly invoices issued by the purchasing dairy company. RMFC was calculated according to Eq. (2):

2 $$RMFC{\text{ }} = {\text{ }}\left( {\rm{average}\,{\text{ }}daily\,{\text{ }}milk\,{\text{ }}yield \times milk\,{\text{ }}price} \right) - \rm{daily}\,{\text{ }}feed{\text{ }}cost$$

The average daily milk yield was obtained from the AMS records, while the milk price corresponded to the amount received by the producer in each month. The daily feed cost was calculated based on the diet formulation and ingredient prices.

In addition to RMFC, the following variables were evaluated: average milk yield (L/cow/day), average daily feed cost (R$/cow/day), and feed efficiency (FE). Milk yield data recorded by the DeLaval DelPro™ system were originally expressed in kg/cow/day and subsequently converted to L/cow/day according to Eq. (1), since milk price and RMFC were expressed on a liter basis.

The average daily feed cost was obtained from the monthly survey of feeds and their respective prices, using purchase invoices recorded in the “feeding expenses” database for LU, LA, and LB. The FE was defined as the ratio between average milk yield (L/day) and estimated dry matter intake (DM; kg DM/day), following the methodology described by Nehme Marinho et al. (2021).

This indicator allows the evaluation of productive performance in relation to feed intake and is essential for analyzing technical and economic efficiency. DM intake was estimated based on diet composition, considering the average dry matter content of each ingredient obtained from laboratory analyses and tabulated values.

### Statistical analysis

Statistical analyses were performed within a comparative observational framework considering herd management group/year (LU–2022, LA–2023, and LB–2023) and month of evaluation. The variables analyzed were milk yield, feed cost, RMFC, and FE. Data were subjected to analysis of variance (ANOVA) after verification of residual normality and homogeneity of variances. Means were compared using Tukey’s test at the 5% significance level. Statistical analyses were performed using AgroEstat® software.

## Results

### Variation of RMFC over the months

The analysis of RMFC over the seven-month period revealed significant differences among the evaluated groups (LU, LA, and LB; Table 5). The LU group, evaluated in 2022, showed higher RMFC values in the final months, reaching R$ 102.21 in August. In 2023, the LA group maintained RMFC values ranging from R$ 63.25 to R$ 81.73, whereas the LB group presented lower values, ranging from R$ 25.64 to R$ 31.04. Statistical analyses confirmed significant differences (*p* < 0.05) both among months within each group and among groups within the same month, according to Tukey’s test.

**Table 5:** Monthly variation in the average revenue minus feed cost (RMFC) of the evaluated groups^1^*

Month of evaluation/group	LA − 2023	LB − 2023	LU − 2022
February	65.30 Aa	65.92 Aa	42.96 Bb
March	68.85 Aa	65.92 Aa	45.76 Bb
April	74.78 Aa	65.55 Ab	41.22 Bc
May	81.73 Aa	55.86 Ac	78.85 Aa
June	63.25 Ab	29.95 Bc	80.11 Aa
July	65.08 Ab	31.04 Bc	101.62 Aa
August	64.58 Ab	25.64 Bc	102.21 Aa

^*^Means followed by different uppercase letters in the same column indicate significant differences among months within the same group. Means followed by different lowercase letters in the same row indicate significant differences among groups within the same month (Tukey’s test, *p* < 0.05)

^1^LA: high-production group; LB: low-production group; LU: single group

### Feed efficiency

The FE, defined as the ratio between milk yield (L/day) and dry matter intake (kg DM/day), varied among groups (Table 6). The LA group showed the highest average FE (1.98), followed by LU (1.57) and LB (1.35). Feed efficiency remained relatively stable across months in LA, while LB exhibited a marked decline from July onward (1.21).

**Table 6:** Average feed efficiency of the groups over the study months (2022 and 2023)^1^

Month	LU 2022	LB 2023	LA 2023
February	1.44	1.39	1.84
March	1.44	1.49	1.95
April	1.51	1.40	1.98
May	1.52	1.39	1.84
June	1.69	1.39	1.84
July	1.69	1.21	1.81
August	1.70	1.21	1.81
Average	1.57	1.35	1.98

^1^LU: Single group; LB: Low-production group; LA: High-production group

### Milk price and total RMFC

**Fig. 1:**
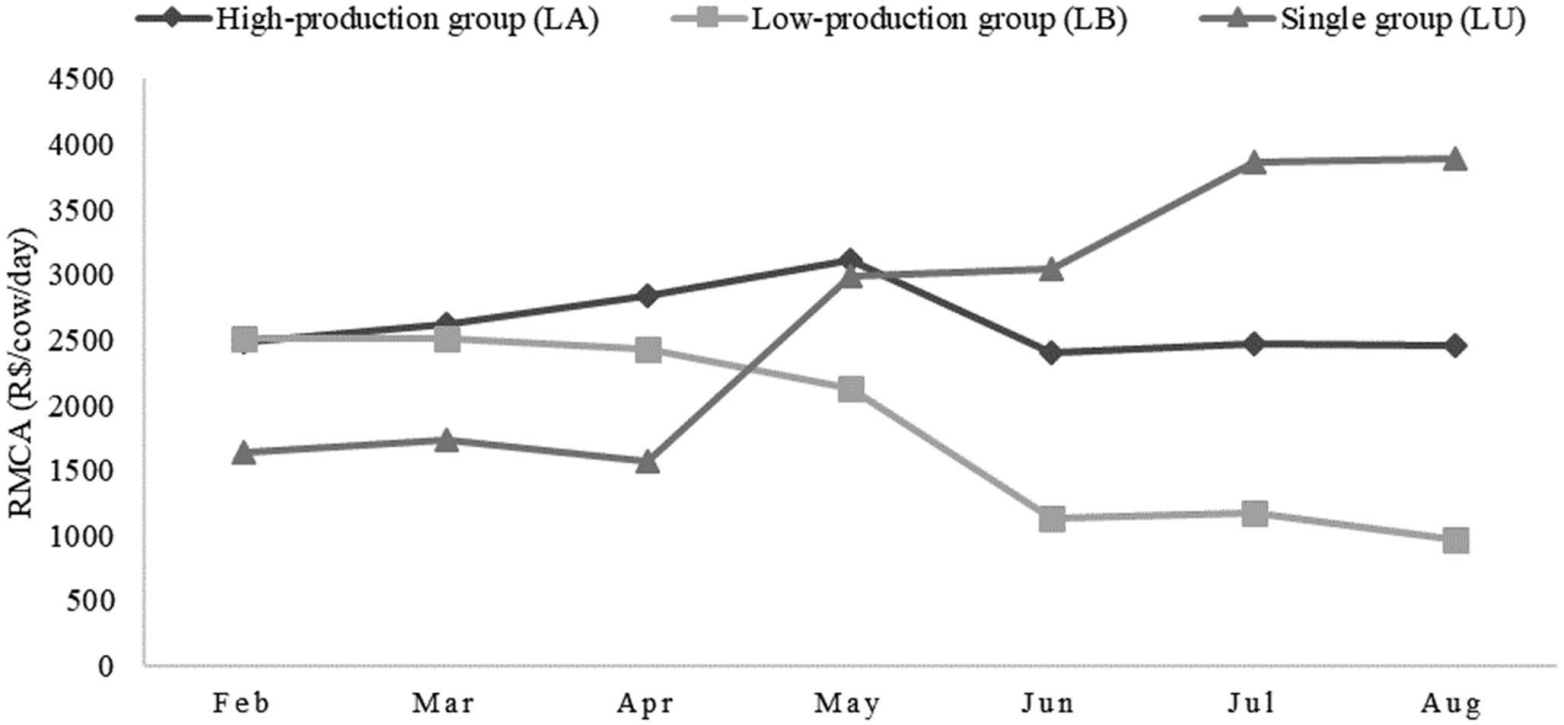
Monthly variation in total revenue minus feed cost (RMFC, in R$) for the three evaluated groups over the study period

Figure 1 illustrates the monthly variation in total RMFC among the evaluated groups. Table 7 presents the milk price (R$/L) received by producers in 2022 and 2023, which directly influenced RMFC values.

**Table 7:** Milk price (R$/L) received by the producer during the study months in 2022 and 2023*

Month	Milk price (2022)	Milk price (2023)
February	R$ 2.43	R$ 2.90
March	R$ 2.43	R$ 2.90
April	R$ 2.43	R$ 3.00
May	R$ 2.98	R$ 2.90
June	R$ 2.98	R$ 2.58
July	R$ 3.49	R$ 2.40
August	R$ 3.81	R$ 2.24

^*^Dairy industry records provided by the partnering milk processor (2022–2023)

## Discussion

The analysis of RMFC over the seven-month period revealed significant differences among the evaluated groups (LU, LA, and LB). The LU group, evaluated in 2022, showed the highest values in the final months, reaching R$ 102.21 in August. However, this result should be interpreted with caution, as RMFC reflects the combined effect of milk yield, feed cost, feeding management, and milk price.

As shown in Table 7, milk price varied substantially across months and between years, ranging from R$ 2.43 to R$ 3.81/L in 2022 and from R$ 2.24 to R$ 3.00/L in 2023. Therefore, the higher RMFC values observed for LU in the final months cannot be interpreted in isolation as evidence that the single-group strategy was economically superior, but rather as the result of the combined productive and economic conditions observed during that period. This interpretation is consistent with Bassotto et al. (2025), who emphasized the sensitivity of economic indicators to fluctuations in milk price. Because LU was evaluated in 2022 and LA/LB in 2023, these differences also reflect year-specific commercial conditions. In addition, it should be considered that the present study was conducted under commercial farm conditions using retrospective production records. Therefore, the observed differences reflect real management and market conditions rather than controlled experimental treatments.

Despite the monthly variation in milk price observed in 2023, the LA group maintained positive and relatively stable RMFC values, ranging from R$ 63.25 to R$ 81.73. This result suggests that the greater productive performance of this group, together with the feeding strategy adopted, was associated with positive economic returns even under higher feed costs. Higher-cost diets may remain economically viable when associated with higher milk yield, reinforcing that lower feed costs do not necessarily translate into better economic performance. This finding is consistent with Britt et al. (2021) and Warner et al. (2020), who reported that high-performing dairy herds may maintain more favorable economic margins even under elevated feeding costs. This result highlights the importance of considering both productivity and feeding strategy when evaluating the economic performance of dairy systems operating under automated milking conditions.

The LB group showed the lowest RMFC values, particularly in the final months of the evaluation period, ranging from R$ 25.64 to R$ 31.04 between June and August. This result is consistent with the lower productive level of this group and suggests reduced economic efficiency under the evaluated conditions. In automated systems, low-producing cows may contribute less to the dilution of feeding and operational costs, which can compromise economic returns. Similar observations were reported by Bach and Cabrera (2017) and Salfer et al. (2017), who highlighted the importance of aligning feeding strategies with productive performance in robotic milking systems.

The monthly decline observed in the LB group also followed the reduction in feed efficiency over time, particularly during the final stage of lactation, which is consistent with Nehme Marinho et al. (2021). In parallel, the different diets adopted for LU, LA, and LB reflected the farm’s attempt to adjust feeding management according to the productive profile of each group. Therefore, the RMFC variation observed in this study should be interpreted as the result of the interaction among production level, feeding management, feed cost, and milk price, rather than as the isolated effect of grouping alone. These findings reinforce that management decisions in automated dairy systems should consider the combined effects of productive performance, feeding strategy, and market conditions in order to optimize economic outcomes.

### Zootechnical performance from the perspective of feed efficiency

FE, defined as the ratio between milk yield (L/day) and dry matter intake (kg DM/day), is widely recognized as one of the main indicators of zootechnical performance in dairy production systems. In systems with high feeding costs, such as automated milking systems, FE plays an even more critical role, as it directly reflects the relationship between nutritional investment and productive return (Atzori et al. 2021).

The analysis revealed substantial differences among the LA, LB, and LU groups, which should be interpreted considering production level, lactation stage, nutritional adequacy, and the management conditions adopted in each evaluated period. The LA group showed the highest average FE (1.98), outperforming both LU (1.57) and LB (1.35). This result is associated with the presence of cows in early lactation and with high daily milk yield, conditions that optimize nutrient utilization. This pattern confirms the findings of Nehme Marinho et al. (2021), who reported increased FE among high-performing cows fed diets adjusted to their productive potential.

The stability of FE in the LA group throughout the months is also noteworthy. The supply of energy-dense diets, including toasted soybean grains, DDG, and a higher proportion of concentrate, contributed to maintaining high milk yield levels (above 48.5 L/cow/day). Such formulations are supported by Kalantari et al. (2016) as essential for groups with higher nutritional requirements, reinforcing the importance of strategic grouping for efficient herd management.

Furthermore, feed efficiency is directly related to the ability to convert nutrients into milk and is influenced not only by lactation stage but also by group homogeneity and the adequacy of the diet to the productive profile. This strategic alignment enables improved productive and economic performance (Bach 2023).

Conversely, the LB group showed a marked decline in FE from July onward (1.21), reflecting the productive limitations of cows in late lactation. This phenomenon can be explained by the physiology of the final third of the lactation curve, when a significant portion of ingested energy is redirected toward body maintenance and gestation rather than milk production (Hurley et al. 2018). The reduced FE observed in this group, even with more economical diets, highlights a productive bottleneck that compromises the profitability of the robotic milking system.

In the LU group, FE showed moderate improvement over the months, increasing gradually from 1.44 in February to 1.70 in August. Under the conditions evaluated in this study, the single-group strategy resulted in lower feed efficiency than the high-production group, although this comparison should be interpreted within the management and commercial context of each year (Cabrera and Kalantari 2016; Rocha-Mendoza et al. 2020). Uniform feeding (a single diet) does not adequately address the physiological heterogeneity of cows, penalizing those in early lactation that cannot fully meet their energy demands, thereby impairing peak yield and increasing body condition loss (Hurley et al. 2018; Nehme Marinho et al. 2021).

Bach (2023) demonstrated that changes in grouping strategies based on lactation stage and productive potential positively affect milk yield and net income, particularly when regrouping is implemented strategically. This approach allows diet adjustment according to the physiological requirements of each group, optimizing zootechnical efficiency and farm profitability.

This type of management segmentation and customization aligns with the organizational innovation strategies described by Devaux et al. (2018), who advocate adaptive and inclusive value chains as pathways toward sustainability and efficiency in emerging economies. Considering the fluctuations in milk prices and their impact on profitability, this scenario also reflects market instability, similar to that identified by Thanh et al. (2022) when analyzing the vulnerability of producers facing economic shocks in developing countries.

In addition, the adoption of Pro-Peak feed in the LA group resulted in significant nutritional gains, increasing diet energy density and directly contributing to improved zootechnical indicators and higher productive efficiency. This nutritional strategy is consistent with the findings of Bach and Cabrera (2017), who highlighted that robotic milking systems enable individualized nutrient delivery, promoting greater economic returns per cow.

Moreover, recent studies have emphasized FE as a key tool for optimizing production, reducing operational costs, and enhancing the sustainability of dairy systems by enabling continuous monitoring of nutrient utilization and performance in real time (Siberski-Cooper and Koltes 2021).

Therefore, feed efficiency is a useful indicator of both individual performance and herd management. When evaluated alongside economic indicators, it enables more accurate diagnostics of nutritional resource utilization, economic sustainability, and the potential for animal culling or regrouping. As noted by Ben Meir et al. (2018), systems that continuously monitor FE tend to respond more rapidly to reproductive, health, and market challenges, ensuring greater economic resilience in the face of volatility in the dairy sector.

In this study, farm profitability was more closely associated with technical efficiency and management conditions than with the absolute number of animals. The milk prices received by the producer varied substantially between months and between years during the study period. As shown in Table 7, prices in 2023 were slightly higher than those in 2022 in the first months but became lower from June onward, reaching R$ 2.24/L in August, whereas the corresponding value in 2022 was R$ 3.81/L. This variation directly affected farm profitability, particularly for the lower-producing group.

According to Grigol (2023), the decline in milk prices received by producers is associated with factors such as increased imports, high industrial stocks, and weakened domestic consumption. This scenario creates downward pressure throughout the value chain, affecting profit margins and discouraging investments in nutrition, genetics, and management key elements for improving feed efficiency and productivity. Moreover, as emphasized by Hanrahan et al. (2018), dairy farm profitability depends not only on production volume but also on efficient technical leverage that optimizes resource use under conditions of market volatility. The present study confirms this relationship: even with greater technical and nutritional efficiency in 2023, profitability was compromised by an unfavorable price environment.

The joint analysis of FE and RMFC allows for a more comprehensive interpretation of both individual and collective herd performance. While RMFC directly reflects the cow’s economic return, FE represents its capacity to convert nutrients into productive output. When assessed together, these metrics help identify productive and economic bottlenecks and support decision-making related to selective culling, regrouping, and nutritional adjustments.

### Limitations

This study has several limitations that should be considered when interpreting the results. First, the analysis was restricted to a single commercial farm in southern Brazil, which limits the extrapolation of the findings to other herd sizes, production systems, and management conditions. Second, the single-group strategy (LU) and the grouped strategy (LA and LB) were implemented in different years; therefore, the observed differences reflect not only herd organization but also year-specific commercial conditions, particularly variations in milk price.

In addition, the grouping strategy, feeding management, and diet formulation were part of the same farm decision-making context and therefore cannot be completely separated analytically. The seven-month study period also represents a relatively short timeframe for evaluating longer-term effects related to lactation stage, seasonal variation, and feed market dynamics. Finally, external factors such as feed ingredient availability, animal health status, and other farm-specific management decisions were not directly controlled.

## Conclusions

This study evaluated the economic performance of two herd management strategies adopted in a guided-flow robotic milking system using RMFC and FE as technical–economic indicators. Under the commercial conditions evaluated, the high-production group (LA) showed higher feed efficiency and more favourable RMFC values than the low-production group (LB), despite requiring greater nutritional investment. In contrast, the low-production group showed reduced technical and economic performance, particularly in the final months of lactation.

The results also indicate that the apparent superiority of the single-group strategy (LU) in some months should be interpreted with caution, since LU was evaluated in 2022, whereas LA and LB were evaluated in 2023 under different milk price conditions. Therefore, the observed differences should be interpreted as the result of the interaction among production level, feeding management, diet cost, and commercial context, rather than as the isolated effect of grouping alone.

Within these limitations, RMFC and FE proved to be useful indicators for describing technical and economic performance under real farm conditions. Future studies should expand the analysis to include more farms, longer evaluation periods, and different robotic milking configurations in order to better distinguish management effects from year-related economic variation and to strengthen the practical interpretation of grouping strategies in automated dairy systems.

## Data Availability

None of the data were deposited in an official repository and are available upon request.
